# Automated informatics may increase the detection rate of suspicious cases of human trafficking—a preliminary study

**DOI:** 10.1093/jamiaopen/ooad097

**Published:** 2023-12-14

**Authors:** David O Duke, Derin Allard, Suzanne Dysart, Keenan O Hogan, Suzanne Phelan, Luke Rawlings, Hanni Stoklosa

**Affiliations:** Arroyo Grande, CA 93421-0266, United States; Family Medicine Residency Program, Marian Medical Center, Santa Maria, CA, United States; Obstetrics and Gynecology, Marian Medical Center, Santa Maria, CA, United States; Pathology and Laboratory Medicine, University of Kansas Health System, Kansas City, KS, United States; Department of Kinesiology and Public Health, California Polytechnic State University, San Luis Obispo, CA, United States; Family Medicine Center, Pacific Central Coast Health Centers, Santa Maria, CA, United States; Department of Emergency Medicine, Brigham and Women’s Hospital, Harvard Medical School, Boston, MA, United States; HEAL Trafficking, Long Beach, CA, United States

**Keywords:** human trafficking, data analysis, electronic health records, patient care team, technology

## Abstract

**Objectives:**

Worldwide, there is an estimated 40.3 million victims trapped in modern day slavery, including 24.9 million in forced labor and 15.4 million in forced marriage. A majority of labor and sex trafficking survivors report at least one healthcare encounter during their victimization. An approach to an informatics technology solution for identifying trafficked persons in real time, in the hospital / emergency department settings is the primary focus of this paper.

**Materials and methods:**

Octavia, a software application implemented in 3 California hospitals, scanned all patient encounters for social and clinical determinants that are consistent predictors of HT. Any encounter that matched these criteria was forwarded to a specially trained High-Risk Navigator who screened the data and when able, made direct contact in an effort to build rapport and possibly provide victim assistance.

**Results:**

During the observation period, the automated scanning of hospital patient encounters resulted in a notable increase in the detection of persons who had a likelihood of being trafficked when compared to a pre-project baseline.

**Discussion:**

Our experience demonstrated that automated technology is useful to assist healthcare providers in identification of potentially trafficked persons, improving the likelihood of care provision.

Key points

- Human trafficking (HT) is a widespread public health concern and merits earnest attention and efforts toward detection, prevention, and intervention.
- Health care systems provide a unique opportunity to provide education and support to persons who may be affected by HT.
- Automated analyses across clinical visit histories can enhance a health system’s efforts at identifying persons who may be victims of HT since some risk factors, patterns, and indicators may be unintentionally missed or overlooked during individual clinical visits.
- Consistent outreach from a single health professional, such as a High-Risk Patient Navigator (HRPN), can foster a relationship of trust with a vulnerable person that may lead to optimal timing for at-risk persons to honestly share their situation or to seek help.

## Background and significance

Around the world, there is an estimated 50 million victims trapped in “modern day slavery,” including 28 million in forced labor and 22 million in forced marriage. Almost one in eight of all those in forced labour are children. More than half of these are in commercial sexual exploitation.^1^ The exact number of victims is largely unknown, as researchers face difficulty in accurately and adequately assessing prevalence and incidence. The interaction with the medical care system may be one of the best opportunities for trafficked persons to be identified and offered assistance. Several studies have shown that up to 80% of trafficked persons have encounters with the health care system during their victimization.^2–12^ In recognition, health care organizations are advocating for and adopting procedures to better identify vulnerable patients and connect them to care.

## Objectives

Analytical tools are commonly used to process categorical risk assessments, such as risk for cardiac event or posthospital discharge complication. Research has explored the use of computational models in discovery and/or tracking of human trafficking (HT).^13–18^ We found no published trafficking research related to the application of computational models in the hospital/emergency department settings specifically. An approach to an informatics technology solution for identifying trafficked persons in real time, in the hospital/emergency department settings is the primary focus of this article.

## Materials and methods

From 2018 to 2022, an application known as “Octavia,” a Whole Person Care software platform, was employed in 3 hospitals of CommonSpirit Health, the largest nonprofit healthcare system in the country. Electronic health record (EHR) data is sent 4 times per hour to Octavia via secure unidirectional API. Octavia organizes these data into a clinically relevant, HIPAA secure shared database for use in direct patient navigational services, outcomes inquiry, and quality improvement analyses. Octavia’s sentry function applies a query rule set to all new incoming data and provides alerts when a match is detected. As the technology piece of the anti-HT team, the Octavia sentry was used to alert a High-Risk Patient Navigator (HRPN) when a match with certain query rule sets or “computational phenotypes” was detected. HRPN are specially trained healthcare professionals, some with lived experience, able to screen for potential abuse, neglect, or violence (ANV), including HT.

The concept of computational phenotype has previously been used in neural and behavioral science, and precision medicine.^19^^,^^20^ In this project, the computational phenotypes consisted of combinations of keywords and other data points that were consistent predictors of persons at-risk of being trafficked (see Table 1). Attributes differed between male and female persons, and included clinical and nonclinical (social determinants of health) attributes, some specific to the geographical region. Intentional effort was made to incorporate additional keywords and diagnoses to ensure inclusion of male patients and those persons who are labor-trafficked, as an omission of labor trafficking in anti-trafficking efforts has been documented in the literature.^21^ Social work notes, care coordinator notes, ICD-10 codes, and emergency department utilization records were mined for keyword matches. Keyword phrases were run retrospectively against results data, and internally validated against identified cases. As results and experiential patterns were observed by the project team, the key word phrases, attributes, and inference logic were continuously adjusted to improve the sensitivity and specificity of the computational phenotype results.

**Table 1.** Computational phenotype keyword/ICD-10 adjustments.

Year	Qtr. 1	Qtr. 2	Qtr. 3	Qtr. 4
**2019**			**Study began**	
**Keyword phrases**			Abdominal, abuse, abusive, anorexia, assault, contusion, dehydration, exploitation, malnutrition, maltreatment, neglect, trauma, O9A.4 Sexual abuse complicating pregnancy, childbirth, and the puerperium, T74.21 Adult sexual abuse, confirmed, T74.22 Child sexual abuse, confirmed, T74.51 Adult forced sexual exploitation, confirmed, T74.52 Child sexual exploitation, confirmed, T74.61 Adult forced labor exploitation, confirmed, T74.62 Child forced labor exploitation, confirmed, T76.12 Current child sexual abuse, T76.21 Adult sexual abuse, suspected, T76.21 XA Adult sexual abuse, suspected, initial encounter, T76.22 Child sexual abuse, suspected, T76.51 Adult forced sexual exploitation, suspected, T76.52 Child sexual exploitation, suspected, T76.61 Adult forced labor exploitation, suspected, T76.62 Child forced labor exploitation, suspected, Y07.6 Multiple perpetrators of maltreatment and neglect, Z04.81 Encounter for examination and observation of victim following forced sexual exploitation, Z04.82 Encounter for examination and observation of victim following forced labor exploitation, Z62.81 Personal history of physical and sexual abuse in childhood, Z62.813 Personal history of forced labor or sexual exploitation in childhood, Z91.4 Personal history of psychological trauma, not elsewhere classified, Z91.41 Personal history of adult abuse- added to this list 7-22-19, Z91.411 Personal history of adult psychological abuse, Z91.412 Personal history of adult neglect, Z91.419 Personal history of unspecified adult abuse, Z91.42 Personal history of forced labor or sexual exploitation, Z91.42 Personal history of forced labor or sexual exploitation, Z91.42 Personal history of forced labor or sexual exploitation, Z91.42 Personal history of forced labor or sexual exploitation	Added: adult abuse, AIDS, Anal, Anus, CWS, Dysuria, Farm worker, Field worker, Force%, Forced labor, gonorrhea, Herpe%, HIV, Laborer, Migrant, neglect, Physical abuse, physical and sexual abuse, psych%, psychological abuse, psychological trauma, rectum, run away, runaway, sex%, Sexual abuse, suicid%, syphilis, traffic%, trans-sexual, Transgender, Transsexual, transvestite, troubled teen, Urinary Tract, vagin%, Violence
**Events**	Late 2018: New ICD-10 Codes Specific for Human Trafficking available for use			ICD-10 searches simplified to key phrase (algorithm can process faster)
**2020**				
**Keyword phrases**	Added: Pelvic, perineal, Perineum, PTSD, Stress Disorder, Transgender, Transsexual, trans-sexual, transvestite Developed Male Specific List: Including penis, testic%, and subtracting nonspecific (for males) phrases such as “black eye”	Added: Rape, Raped, Rapped (sp), Molestation, Molested		Added: Pushed by, struck by husband, struck by wife, struck by spouse, struck by boyfriend, struck by girlfriend, struck by significant other, struck by SO
**Events**		COVID staffing shortages; HRPN pulled for COVID response		
**2021**			**Study ended**	
**Keyword phrases**		Added: Safe House		
**Events**	First COVID Vaccinations; HRPN pulled to alert patients of COVID results and track contacts			

The elements of the computational phenotypes are supported in the current limited body of literature reporting on key characteristics of trafficked persons (adults and minors), including unstable disposition, signs of abuse/trauma, malnutrition/dehydration, uro-gynecologic complaints, sexually transmitted infections, suicidal ideation/attempts, mental health disorders, and increased health care utilization.^22–27^ Populations that are most vulnerable to HT are children in the welfare, foster care, or juvenile justice systems, and people of all ages who experience housing insecurity. High-risk populations additionally include undocumented immigrants, indigenous peoples, migrant laborers, persons with limited English proficiency, persons with disabilities, people who identify as LGBTQI+, and individuals with substance use disorders or in rehabilitation programs within the criminal justice system.^28–36^ Often, at-risk cohorts fall into several of these categories at once.

Upon receiving an alert, the HRPN screened the clinical and social data available and when able, made direct contact with the person. To maximize development of rapport and trust, effort was made to provide longitudinal support—consistent contact with the same HRPN upon every subsequent clinical visit following the visit which prompted the initial alert.

## Results

The observational period for the CommonSpirit Health Central Coast project included 4th quarter 2019 through 3rd quarter 2021. The Octavia sentry alerted to an average of 1 to 8 potential cases daily (out of an average of 440 total daily encounters) for HRPN review. About 43.17% of the alerts were reviewed by the HRPN. Of those reviewed, 24% of cases were considered “highly suspicious” or known confirmed cases of HT, totaling 184 during the 23-month observational period. Comparatively, as part of CommonSpirit Health’s commitment to care and protection of trafficked persons, and in support of a grant proposal to the United States Department of Justice, preproject baseline data was released for 2017-2018 that included 10 total patients per year identified as “possible” cases of ANV including HT in the 3 Central Coast hospitals.

Resourcing of a HRPN during this observational period was affected by the COVID pandemic response with high hospital census combined with limited staffing, requiring the HRPN to divert time from the anti-HT project to direct clinical patient care. This is reflected in the 47.3% alert response, with no alert response in September 2020 (see Table 2).

**Figure 1.**
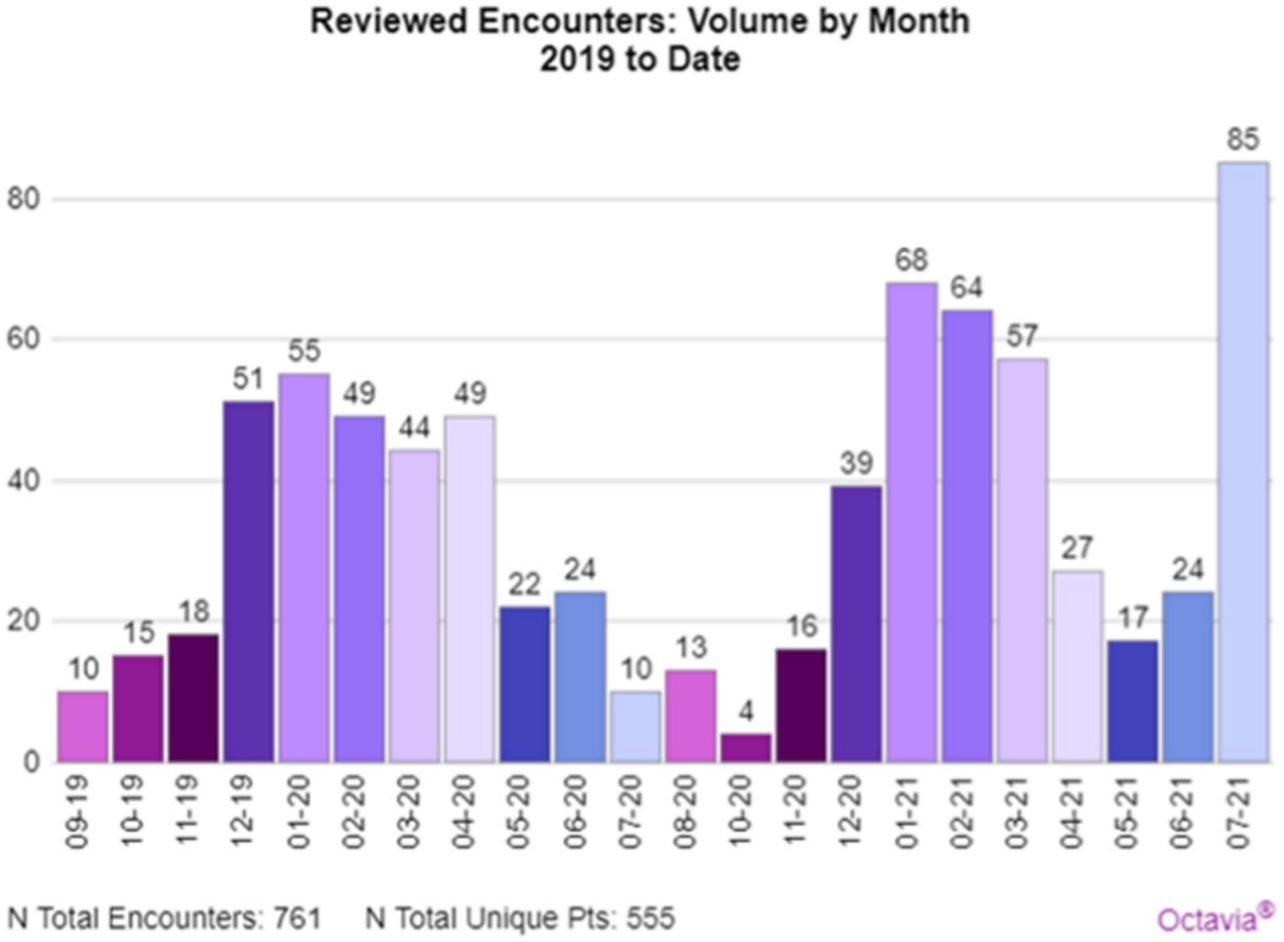
High Risk Patient Navigator number of cases reviewed. Bar colors provide visual differentiation in months. This graph depicts fluctuation in available capacity of navigators to review encounters over the course of the program to date. Dips in Navigator’s capacity are attributed to other priorities (eg, COVID-19 support) and demands (paid leave and etc.) during the period suggesting a single Navigator was not able to review all alerts, all of the time.

**Figure 2.**
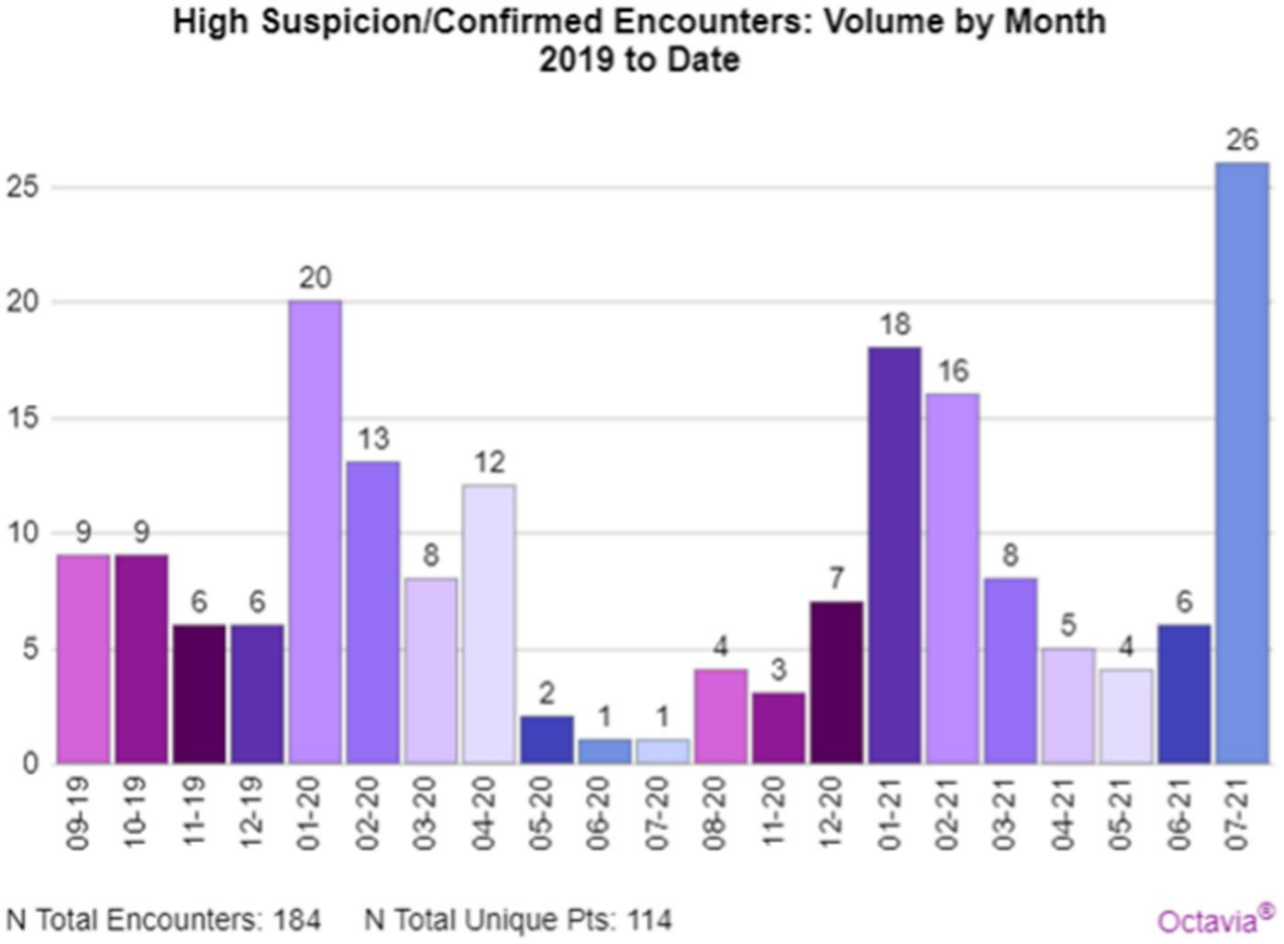
Detection (number of cases determined “highly suspicious”).

Figure 1 illustrates the number of cases reviewed by the HRPN per month in response to Octavia sentry alerts. Figure 2 depicts the number of the cases reviewed that were determined by the HRPN to be “highly suspicious.”

**Table 2.** Results of Octavia alert volume, case review, and identification.

Baseline: Pre-Octavia					**No. of “possible”** **cases of HT**	
	2017				10	
	2018				10	

Octavia test period		**Octavia alerts**	**HRPN screened**	**% alerts screened**	**No. of “highly suspicious”**	**% screened in**
	September to December 2019	276	94	34.06	30	31.91
	2020	838	325	38.78	71	21.85
	January to July 2021	649	342	52.7	83	24.27
	**Total**	**1763**	**761**	**43.17**	**184**	**24.18**

## Discussion

While the preliminary work during this observational period shows a promising process in identifying trafficked persons, it is acknowledged that limitations exist. Limitations of the presented work and its subsequent conclusions include the small sample size accrued from 3 mid-sized California hospitals. The population of HT victims in California who accessed health care in these systems in the study time frame, while large, cannot be presumed representative of all patients experiencing HT. The computational phenotype keyword dataset is iteratively being improved and some biases have been identified. One limitation in early iterations was the words “lesbian,” “gay,” “bisexual,” and “homosexual” were not included in the computational phenotype keyword dataset. Additionally, it must be recognized that the identification, assessment, and policy making regarding sex trafficking are disproportionately greater than that directed toward labor trafficking.^21^ As such, it is possible that our methods of identification may have also been biased toward criteria consistent with sex trafficking.

The determination of a case to be “highly suspicious” was ultimately a subjective decision of the HRPN, based on the information available at the time. This subjectivity persists until a case is confirmed by either self-report or law enforcement report. Despite active follow-up by care teams, the vast majority of at-risk cases remain classified as “highly suspicious” without the potential victim clearly confirming and/or agreeing to formally report their case. It is not possible to know the true denominator of HT cases passing through our facility doors thus positive predictive value calculation is ultimately limited.

The practice of patient profiling in healthcare, including concerns regarding the possible stigmatization of vulnerable populations, has been raised. With inclusion of a wide breadth of elements, not confined to the attributes most often associated with profiling (including race, gender, disability, social class), the unintended consequences of discrimination or stigmatization of any single population hopes to be mitigated. The Center for Disease Control’s has added “suspected human trafficking” diagnosis codes (eg, ICD-10 code *T76.5—Forced sexual exploitation, suspected*) to the International Classification of Diseases (ICD).^37^ Although Octavia does look for these codes in the diagnoses, it is worth considering that adding these diagnoses or verbiage to the medical chart without timely patient knowledge might put the patient at risk if an abuser finds this information on the discharge paperwork and medical records. This concern is further magnified by the recent implementation of the information blocking provisions of the 21st Century CURES Act, which penalizes the delayed transmission of medical documentation to patients outside of narrowly defined exceptions.^38^ As immediate electronic access to patient medical documentation becomes mainstream, patients will likely face increased risk in seeking medical attention due to the *de facto* access maintained by abusers and traffickers via coercion and violence. Individual hospital administrations are responsible for developing and implementing policies compliant with these regulations, balancing medical transparency and patient safety, which may be greatly benefited by automated alerting and tracking of a high-risk patient population.

While safeguards were in place to prevent bias, researcher affiliation with the software company that produces the Octavia application (researchers D.O.D. and K.N.) could have led researchers to inadvertently misattribute outcomes as due to the software versus other factors (measured or unmeasured). While independent researchers unaffiliated with the software company were involved in all phases of the project and gave approval for final manuscript submission, their collaboration with team members involved in the software company could have nevertheless introduced bias.

## Conclusion

Per the U.S. Department of State, data and statistics may not reflect the full scope of the forced labor problem, due to the hidden nature of the crime, challenges in identifying individual victims, gaps in data accuracy and completeness, and significant barriers regarding the sharing of victim information among various stakeholders.^39^ Thus, efforts to increase the detection of highly suspicious cases of HT, and the offer of services in a safe, trauma-informed environment, particularly from someone with whom the person has developed a rapport, may be a worthy strategy to pursue. It is our hope that this preliminary study will encourage further exploration in the use of automated technology to improve the detection of possible trafficked persons, as well as consideration of the merits of providing longitudinal, rapport-developing support in a healthcare setting. Additionally, we advocate for trauma-informed health system policies that connect people experiencing trafficking to the provision of longitudinal services. These may include connecting trafficked patients to housing as well as access to specialty medical clinics dedicated to the follow-up and care with a requirement that all providers working in the clinic receive the appropriate training, support, and resources. Trauma-informed specialty clinics have already been implemented in the CommonSpirit Health System (Fresno, CA) and are demonstrating promising results. The CA Central Coast hospitals that sponsored this project launched a specialty clinic in the autumn of 2022.

## Data Availability

Data are available on request only due to ethical, legal, or commercial reasons.
